# Effect of various surface treatments on the shear bond strength of 3D-printed denture base resins

**DOI:** 10.6026/973206300210813

**Published:** 2025-04-30

**Authors:** Pooja Arora, Suni Kar, Jasmine Marwaha, Shubham Bhuktar, Akash Raj Sharma, Abesh Kumar Jana

**Affiliations:** 1 Department of Prosthodontics, Faculty of Dentistry, Taif University, Taif, Saudi Arabia - 21944; 2 Department of Prosthodontics, Hitech Dental College, Bhubaneswar, Odisha- 751025, India; 3 Department of Conservative dentistry and Endodontics, Maharishi Markandeshwar college of dental sciences and research, Mullana, Haryana, India - 133203; 4 Department of prosthodontics and crown and bridge and implantology, Dr. Rajesh Ramdasji Kambe Dental college and hospital, Kahneri Sarap, Akola-444401, Maharashtra, India; 5 Department of Prosthodontics and Crown & Bridge, Subharti Dental College & Hospital, Swami Vivekanand Subharti University, Meerut-250003, Uttar Pradesh, India; 6 Department of Oral Medicine and Radiology, Kalinga Institute of Dental Sciences, 'KIIT Deemed to be University', Patia, Bhubaneswar, Odisha, India

**Keywords:** 3D-printed denture base resin, shear bond strength, surface treatment, plasma treatment, air abrasion, silica coating

## Abstract

Evaluation of various surface treatments is of interest to measure Shear Bond Strength (SBS) in 3D-printed denture base resins. Hence, sixty specimens were organized into control and three experimental groups: air abrasion, silica coating, and plasma treatment. Plasma treatment produced the strongest SBS result of 14.3 ± 1.2 MPa but the control group achieved only 5.2 ± 0.8 MPa (p < 0.001). The adhesion increased substantially after surface treatments but plasma treatment demonstrated the highest level of success. Thus, modifying 3D-printed denture bases through surface treatments allows for better performance in clinical settings.

## Background:

3D printing technology in dentistry made possible the manufacture of denture base resins through improved accuracy while reducing materials usage and enhancing product customization for dental patients [1]. The essential bond quality between 3D-printed denture bases and veneering materials is a main clinical issue because weak attachment leads to significant functional failures like material separation and fracturing [2, 3]. The bonding force known as shear bond strength directly influences the long-term durability of dentures made from these materials. The bonding efficiency of denture base resins improves through surface modifications that modify their surface roughness and wettability according to studies [4, 5]. The bond strength results from mechanical interlocking or chemical adhesion through established surface treatments that use air abrasion, silica coating and plasma treatment [6, 7]. Shear bond strength of 3D-printed denture base resins is significantly lower than that of conventional and milled resins, highlighting repair challenges with digital materials [8]. Therefore, it is of interest to investigate the influence of numerous surface finishing techniques on SBS levels between 3D-printed denture base resins and veneering materials.

## Materials and Methods:

## Specimen preparation:

The framework fabricators used 60 disk-shaped specimens with 10 mm diameter and 2 mm thickness from NextDent^TM^ Denture 3D+ manufactured by NextDent in Netherlands. The printer operated under digital light processing guidelines to produce the specimens after their post-processing phase involved isopropyl alcohol rinsing and curing with ultraviolet light to achieve total resin polymerization.

## Surface treatment groups:

A total of 60 3D-printed resin specimens received one of four distinct surface treatments according to a random partition of the specimens into four groups with 15 examples each. Group A served as the control group because the researchers applied no surface treatments. The treatment applied to Group B included air abrasion of specimens with 2 bar aluminium oxide particle pressure for 10 seconds at a surface distance of 10 mm. The specimens in Group C received a tribochemical silica coating by using CoJet^TM^ (3M ESPE) with 2.5 bar pressure for 15 seconds before performing a subsequent silane application. Surface energy enhancement took place when specimens underwent 60-second oxygen plasma treatment at 50 W from Femto Science based in South Korea for improved adhesion.

## Bonding procedure:

A 4 mm diameter and 3 mm height cylindrical mold received the resin-based veneering material known as GC Gradia^TM^ from GC Corporation Japan. Veneering resin received polymerization for 40 seconds through the Ivoclar Vivadent Bluephase G2 LED light-curing unit operated at 1200 mW/cm^2^ according to manufacturer suggestions.

## Shear bond strength testing:

All bonded specimens underwent a 37°C water immersion at 37°C for 24 hours before completion of testing. The shear bond strength (SBS) assessment occurred on the Intron 3345 universal testing machine (USA) by applying a 1 mm/min crosshead speed for failure occurrence. The testing instrument measured debonding force as Newtons (N) which served as input for computing shear bond strength from the following formula:

SBS (MPa) =Force (N) Bonded area (mm2) SBS (MPa) = Bonded area (mm2) Force (N)

## Failure mode analysis:

Under a 20x magnified stereomicroscope (Olympus SZ61, Japan) researchers examined and determined the failure modes of tested specimens as adhesive or cohesive or both.

## Statistical analysis:

One-way ANOVA with Tukey's post-hoc test (α=0.05) analyzed the values to identify significant differences between groups. All statistical evaluations took place through SPSS software version 26.0 which IBM Corp. USA provides.

## Results:

The shear bond strength (SBS) values for the different surface treatment groups exhibited significant variations. The highest mean SBS was observed in the plasma-treated group (Group D), while the lowest was recorded in the control group (Group A). A statistically significant difference was found among all groups (p < 0.05). The mean SBS values along with standard deviations for each group are summarized in Table 1. The SBS values (in MPa) for the four groups were as follows: Group A (Control) had the lowest SBS (5.2 ± 0.8 MPa), followed by Group B (Air Abrasion) with an intermediate SBS (9.1 ± 1.0 MPa). Group C (Silica Coating) demonstrated a higher bond strength (11.5 ± 1.3 MPa), while Group D (Plasma Treatment) exhibited the highest SBS (14.3 ± 1.2 MPa). The statistical analysis confirmed significant differences among the groups (p < 0.001). Failure modes were categorized as adhesive, cohesive, or mixed. The control group (Group A) exhibited predominantly adhesive failures (80%), while Groups B and C showed mixed failure patterns (60% and 70%, respectively). The plasma-treated specimens (Group D) had the highest proportion of cohesive failures (75%), indicating a stronger bond between the denture base resins and veneering material (Table 2). The plasma-treated group demonstrated the highest SBS, which was significantly greater than the control and air-abraded groups (Table 1). Moreover, the failure mode analysis suggested that plasma treatment enhanced cohesive bonding (Table 2). These findings indicate that modifying the surface of 3D-printed denture base resins improves adhesion to veneering materials, with plasma treatment being the most effective method.

## Discussion:

This research investigated which different surface treatments deliver the strongest shear bond strength (SBS) results for 3D-printed denture base resins. Plasma treatment produced the greatest bond strength results compared to silica coating then air abrasion and the baseline had the weakest bond strength. Surface modifications become essential because they improve the adhesive properties between veneering materials and 3D-printed denture bases. The bond strength of denture base resins depends heavily on the surface condition of roughness and wettability. The surface enhancement technique of air abrasion brings increased roughness to the material which helps create micromechanical interlocking bonds [1, 2]. The impact of air abrasion on 3D-printed resins depends on their specific material makeup in addition to the parameters used during printing processes. Air-abraded specimens demonstrated stronger bond strength than control samples while not producing chemical bonds with veneering resin according to previous research [3, 4]. The tribochemical silicone coating treatment enhanced SBS by achieving mechanical attachment as well as chemical bond formation sites [5, 6]. The repairability of 3D printed denture base polymers is influenced by both surface treatment and artificial aging, with untreated aged surfaces showing significantly reduced shear bond strength [7]. Surface treatments, particularly sandblasting, significantly enhance the bond strength across all resin types, making them essential for effective denture repairs regardless of fabrication method [8, 9] and this result corresponds with the present study. Plasma surface treatment produced the strongest SBS values in comparison to every other group investigated here. Plasma surface modification changes resin chemical composition as well as surface energy which make the resin more wettable for better adhesive bonds [10, 11]. Despite clinical acceptability for complete dentures, additional retention strategies are advised when using printed denture bases in implant-retained prostheses due to weaker bond performance [12]. Significant variability exists among 3D printed resins, with each brand displaying distinct surface and mechanical characteristics, underscoring the need for material-specific performance evaluations [13, 14]. 3D-printed denture base resins exhibit significantly lower flexural strength, impact resistance, and hardness compared to heat-polymerized resins, though they offer smoother surface finishes. Thermal cycling further degrades the mechanical properties of 3D-printed resins, emphasizing the need for durability considerations in long-term clinical applications [15].

## Conclusion:

Prosthetic success depends heavily on the strong bond which develops between denture base resins and veneering materials. Plasma treatment applied to 3D-printed resins improves their adhesive qualities which helps prevent delamination failure. However, studies are required to measure system functions when used under oral conditions that are exposed to thermo cycling.

## Figures and Tables

**Table 1:** Shear bond strength of 3D-printed denture base resins after different surface treatments

**Group**	**Surface Treatment**	**Shear Bond Strength (MPa) (Mean ± SD)**
Group A	Control (No Treatment)	5.2 ± 0.8
Group B	Air Abrasion	9.1 ± 1.0
Group C	Silica Coating	11.5 ± 1.3
Group D	Plasma Treatment	14.3 ± 1.2
(p < 0.001,
statistically significant among groups)

**Table 2:** Failure mode distribution among different surface treatment groups

**Group**	**Surface Treatment**	**Adhesive Failure (%)**	**Cohesive Failure (%)**	**Mixed Failure (%)**
Group A	Control (No Treatment)	80	10	10
Group B	Air Abrasion	40	30	30
Group C	Silica Coating	25	35	40
Group D	Plasma Treatment	10	75	15
